# Rock glaciers in crystalline catchments: Hidden permafrost‐related threats to alpine headwater lakes

**DOI:** 10.1111/gcb.13985

**Published:** 2017-12-04

**Authors:** Boris P. Ilyashuk, Elena A. Ilyashuk, Roland Psenner, Richard Tessadri, Karin A. Koinig

**Affiliations:** ^1^ Institute of Ecology University of Innsbruck Innsbruck Austria; ^2^ Institute for Alpine Environment Eurac Research Bozen/Bolzano Italy; ^3^ Institute of Mineralogy and Petrography University of Innsbruck Innsbruck Austria

**Keywords:** acid drainage, chironomid deformities, cryosphere, ecotoxicology, frost action, metals, paleolimnology, periglacial environments

## Abstract

A global warming‐induced transition from glacial to periglacial processes has been identified in mountainous regions around the world. Degrading permafrost in pristine periglacial environments can produce acid rock drainage (ARD) and cause severe ecological damage in areas underlain by sulfide‐bearing bedrock. Limnological and paleolimnological approaches were used to assess and compare ARDs generated by rock glaciers, a typical landform of the mountain permafrost domain, and their effects on alpine headwater lakes with similar morphometric features and underlying bedrock geology, but characterized by different intensities of frost action in their catchments during the year. We argue that ARD and its effects on lakes are more severe in the alpine periglacial belt with mean annual air temperatures (MAAT) between −2°C and +3°C, where groundwater persists in the liquid phase for most of the year, in contrast to ARD in the periglacial belt where frost action dominates (MAAT < −2°C). The findings clearly suggest that the ambient air temperature is an important factor affecting the ARD production in alpine periglacial environments. Applying the paleoecological analysis of morphological abnormalities in chironomids through the past millennium, we tested and rejected the hypothesis that unfavorable conditions for aquatic life in the ARD‐stressed lakes are largely related to the temperature increase over recent decades, responsible for the enhanced release of ARD contaminants. Our results indicate that the ARDs generated in the catchments are of a long‐lasting nature and the frequency of chironomid morphological deformities was significantly higher during the Little Ice Age (LIA) than during pre‐ or post‐LIA periods, suggesting that lower water temperatures may increase the adverse impacts of ARD on aquatic invertebrates. This highlights that temperature‐mediated modulations of the metabolism and life cycle of aquatic organisms should be considered when reconstructing long‐term trends in the ecotoxicological state of lakes.

## 1 INTRODUCTION

Earth's surface temperatures are close to the triple point of water (0.01°C), the temperature at which liquid water, solid ice, and water vapor coexist in thermodynamic equilibrium (Marshall, 2012). The cryosphere, which encompasses those portions of the Earth's surface where water is in solid form (ice) for at least part of the year, is very sensitive to changes in global mean temperature due to its proximity to melting conditions. It is one of the most dynamic components of the Earth system, since any imbalance of the thermodynamic equilibrium leads to either growing or shrinking ice masses (Marshall, 2012). In mountain areas, the rate of warming is amplified with elevation, and high alpine environments experience more rapid changes in temperature than environments at lower elevations (Acquaotta, Fratianni, & Garzena, 2015; Pepin et al., 2015). As a consequence, components of the mountain cryosphere (glaciers, mountain permafrost, seasonally frozen ground, ice on lakes, etc.) in high‐elevation areas are extremely sensitive to changes in global climate.

Since the termination of the Little Ice Age (LIA; ca. middle of 19th century), alpine glaciers in most parts of the Northern Hemisphere have retreated almost to their minimum extent during the Holocene thermal maximum (Solomina et al., 2015) between 11,000 and 5,000 years ago (Renssen et al., 2009). Particularly, high rates of glacier retreat and shrinkage are observed in the early 21st century (Zemp et al., 2015). A global warming‐induced upward shift in the equilibrium line altitude (ELA) of glaciers results in a transition from glacial to periglacial processes in recently deglaciated forefields, namely those once occupied by LIA glaciers. As a consequence, a recent or ongoing transition from glacial to periglacial landforms has been identified in many mountain areas around the world. For example, the evolution of typical glaciers to debris‐covered glaciers and eventually to rock glaciers, that is ice/debris mixtures creeping downslope under gravity in permafrost conditions (Berthling, 2011), has been described in many high‐elevation regions, such as the Himalaya in Asia (Shroder, Bishop, Copland, & Sloan, 2000), the American Cordillera (e.g., Emmer, Loarte, Klimeš, & Vilímek, 2015; Monnier & Kinnard, 2015), the Pyrenees (Lugon et al., 2004), the Sierra Nevada (Gómez‐Ortiz et al., 2014), and the Alps (e.g., Bosson et al., 2015; Dusik et al., 2015; Seppi et al., 2015) in Europe. In the meantime, ice in rock glaciers and other landforms that contain ice‐rich permafrost is also melting because of warming and the concomitant upward shift of ELA (Haeberli & Gruber, 2009). The expansion and upward movement of the periglacial mountain belts in response to climate warming are usually accompanied by an increase in runoff from alpine basins (Casassa, López, Pouyaud, & Escobar, 2009) and rapid formation and growth of lakes and ponds in deglaciating areas (Tartari, Salerno, Buraschi, Bruccoleri, & Smiraglia, 2008; Salerno et al., 2014), which are an important component of alpine periglacial landscapes.

Despite the fact that changes in the mountain cryosphere are progressing rapidly, surprisingly little research has been devoted to study the impact of meltwater from glaciated and perennially frozen areas on alpine aquatic ecosystems (Slemmons, Sarosa, & Simon, 2013; Sommaruga, 2015). In recent years, an increasing number of studies have documented that climate change is causing rising solute concentrations in mountain lakes and streams, especially in areas characterized by glaciers, rock glaciers, or other cryospheric features (e.g., Barnes, Williams, Parman, Hill, & Caine, 2014; Salerno et al., 2016; Thies et al., 2007). Carbonate dissolution and sulfide oxidation can account for up to 90% of the solute flux from newly exposed rocks to air and oxygenated water by retreating glaciers or thawing permafrost (Anderson, Drever, Frost, & Holden, 2000).

Extremely serious environmental problems associated with the generation of acid rock drainage (ARD) can arise in mineralized watersheds with sulfide‐bearing bedrock. Changes in the location of glacier ice and ice‐rich permafrost can greatly affect ARD in areas where the ice has been providing a cover to prevent oxidation of sulfide minerals (Dold et al., 2013; Fortner et al., 2011; González‐Toril et al., 2015; Mast, Turk, Clow, & Campbell, 2011; Todd et al., 2012). Furthermore, the oxidation rate of sulfide minerals is strongly temperature‐dependent and increases with temperature (Ahonen & Tuovinen, 1991; Schoonen, Elsetinow, Borda, & Strongin, 2000). ARD produces acidic waters rich in sulfuric acid and metals that adversely affect the environment and living organisms. Many metal ions form complexes with various organic ligands, which can be easily transported across cell membranes (Argüello, Raimunda, & González‐Guerrero, 2012) and cause mutagenic and cytotoxic effects on living organisms, even at low concentrations (Planelló, Martínez‐Guitarte, & Morcillo, 2007). It is impossible to identify the toxic effects of specific metals in natural environments where organisms are exposed to a large number of different metals at the same time. Whenever metal ions are present in a mixture, they interact, producing additive, synergistic or antagonistic effects on exposed organisms (Sprague, 1985). Despite the widespread documentation of metal contamination in aquatic ecosystems, relatively little is known about the long‐term impacts of metal exposures on populations of aquatic organisms, especially on multi‐decadal to centennial time scales through past millennia (Korosi, Thienpont, Smol, & Blais, 2017a, b).

As of today, only a few studies exist which have been dealing with the investigation of ARD in periglacial environments (Downing & Jacobs, 2014 and references therein). Periglacial areas are exposed to cold, non‐glacial conditions with frost action, and the +3°C isotherm of mean annual air temperature (MAAT) can be used as a boundary between periglacial and non‐periglacial areas (French, 2007; Williams, 1961). A key feature of alpine periglacial environments is the phase change of water from liquid to solid and vice versa, caused by repeated diurnal and seasonal freeze–thaw cycles (French, 2011). Frost action in bedrock, generally associated with volumetric expansion of ice and/or ice segregation, leads to mechanical disintegration of rocks and an increase in fresh rock surfaces exposed to oxidative weathering. A volumetric expansion of approximately 9% accompanying the freezing of water has traditionally been assumed to be the main causal mechanism of rock fracture (Hall, Thorn, Matsuoka, & Prick, 2002), though recently the role of ice segregation, namely the formation of ice lenses in rocks as a result of the migration and subsequent freezing of pore water, has been recognized as potentially more significant (Murton, Peterson, & Ozouf, 2006). Frequent and prolonged wetting of mineral surfaces by meltwater tends to generate a more constant volume of acids and other contaminants as water moves through, and flushes oxidation products out of, a drainage system (Lottermoser, 2010). Recent investigations in an alpine watershed with sulfide‐bearing lithology have demonstrated that rock glaciers, one of the most common permafrost landforms in periglacial mountain environments, can represent potent sources of acidic, metal‐rich solutes that severely disturb lake ecosystems (Ilyashuk, Ilyashuk, Psenner, Tessadri, & Koinig, 2014; Thies et al., 2007). The incidence of morphological abnormalities (response at the phenotypic level) in chironomid populations inhabiting such lakes can be as high as that recorded in chironomid populations from sites heavily polluted by trace metals of anthropogenic origin (Ilyashuk et al., 2014).

**Figure 1:**
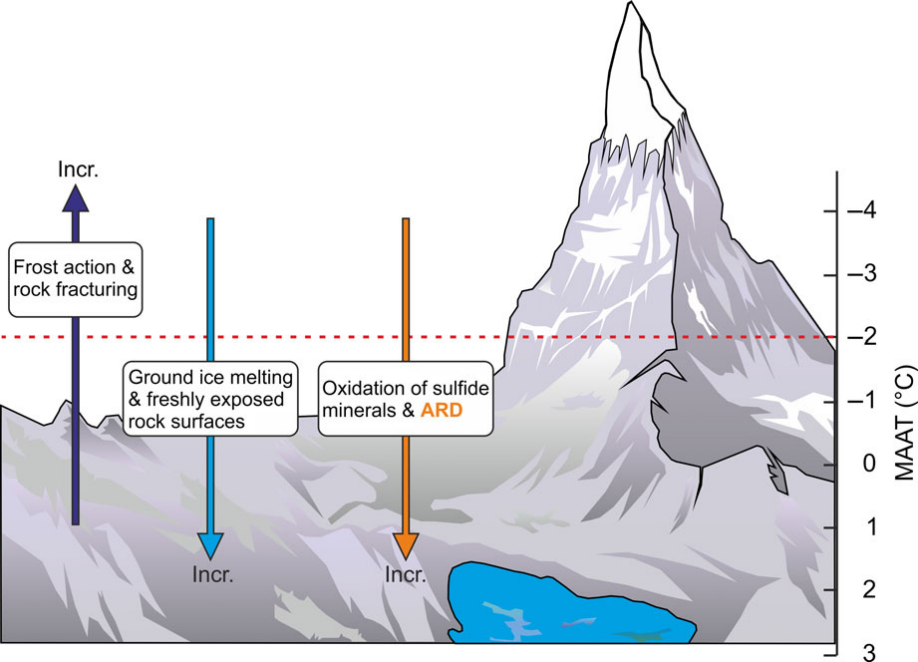
Conceptual model of acid rock drainage generation in alpine periglacial environments. The dashed line indicates the −2°C isotherm of mean annual air temperature (MAAT), which subdivides the periglacial belt into environments in which frost action conditions dominate (MAAT lower than −2°C) and those in which groundwater persists in the liquid phase for most of the year and frost action occurs but does not dominate (MAAT between −2°C and +3°C) (French, 2007, 2011) [Colour figure can be viewed at wileyonlinelibrary.com]

The periglacial belt, in turn, is subdivided by the −2°C isotherm of MAAT into environments in which frost action conditions dominate (MAAT lower than −2°C) and those in which groundwater persists in the liquid phase for most of the year and frost action occurs but does not dominate (MAAT between −2°C and +3°C) (French, 2007, 2011). This implies that the processes of rock fracture and formation of freshly exposed rock surfaces dominate in areas with a MAAT lower than −2°C, whereas the oxidative weathering of sulfide minerals and the generation of ARD proceed more intensively in areas with a MAAT of between −2°C and +3°C, where water remains in the liquid state for longer periods of time (Figure 1). Taking into account these differences, we hypothesized that ARD generated by a rock glacier and its effects on a lake in a crystalline‐rock area are more severe in the alpine periglacial belt in which groundwater persists in the liquid phase for most of the year, in contrast to the periglacial belt in which frost action conditions dominate.

The present study aimed at testing this hypothesis by assessing and comparing ARDs generated by rock glaciers and their effects on two alpine headwater lakes with similar morphometric features and bedrock geology, but characterized by different intensities of frost action in their catchments. As a reference, we sampled a third high alpine lake, not influenced by rock glaciers and without obvious signs of ARD in the catchment. The ARD effects were assessed through the determination of the concentrations of metals in water, sediment, and biota of the lakes, and the frequency of morphological deformities in chironomids. We also hypothesized that, due to reduced oxidation of the sulfide‐bearing minerals at lower temperatures, conditions for aquatic life in lakes affected by ARD may have been more favorable during colder climate phases than during warmer phases in the past. In this regard, we applied paleolimnological techniques to track and compare changes over the past millennium in the incidence of morphological abnormalities in populations of a chironomid species inhabiting all three lakes.

## 2 MATERIALS AND METHODS

### 2.1 Study area

The study area is located within a crystalline‐rock watershed in periglacial environments of the upper Vinschgau valley in the Central Eastern Alps, South Tyrol, Italy. This region of the European Alps is known for a high rock glacier density. The latest rock glacier inventory of South Tyrol resulted in the identification of 240 active and 59 inactive rock glaciers (Monreal & Stötter, 2010). The Vinschgau is a typical inner‐Alpine valley characterized by a dry climate with a total annual precipitation below 600 mm (Grashey‐Jansen & Schröder, 2009; ZAMG, 2015). High solar radiation and low humidity determine a high potential evapotranspiration that exceeds precipitation by 400–500 mm (Otto, 1974). The bedrock geology of the valley belongs to the Austroalpine Oetztal‐Stubai crystalline massif, a polymetamorphic terrain composed of orthogneisses, paragneisses, and schists (Tollmann, 1980). The rocks are characterized by veins filled with quartz, ankerite, and sulfide ore minerals, most notably pyrite and chalcopyrite (Potro, 1982; Sonnleitner, Redl, & Schinner, 2011). These disseminated sulfide ore minerals are widespread in the bedrock (Koltai, Spötl, Luetscher, Barrett, & Müller, 2017; Spötl, Unterwurzacher, Mangini, & Longstaffe, 2002) and are undoubtedly one of the key factors in generating ARDs in the study area.

**Figure 2:**
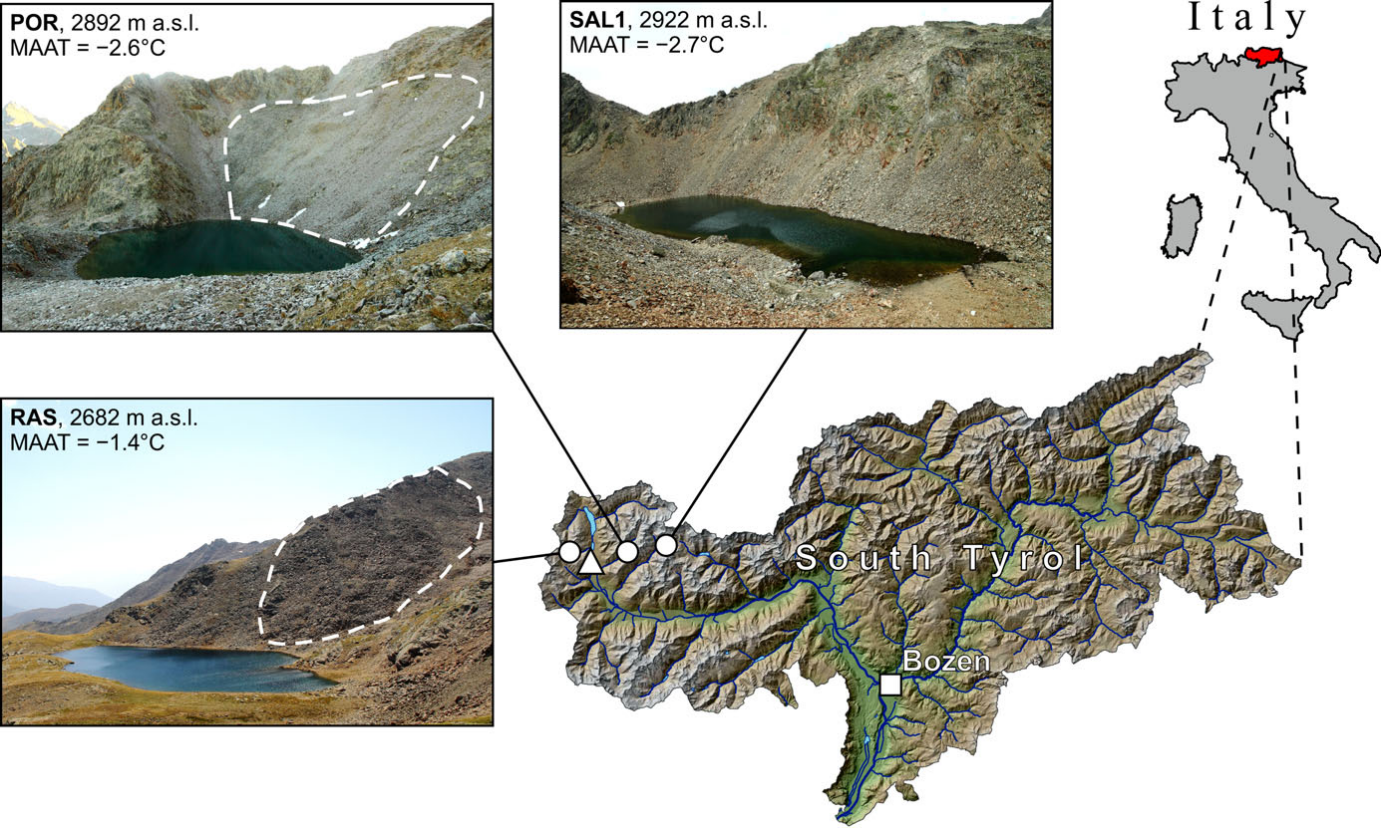
Overview maps showing the location of the study area and photographs of the study lakes and their catchments. Lakes (white circles): POR = Portles See, SAL1 = Oberer Saldursee, and RAS = Rasass See; MAAT = mean annual air temperature. The position of the weather station Marienberg/Montemaria is indicated by the white triangle. The white dashed lines indicate the boundaries of the rock glaciers. The maps are adapted from http://d-maps.com/ [Colour figure can be viewed at wileyonlinelibrary.com]

A comparative study of three remote high alpine lakes with relatively similar morphometric features was conducted in 2010–2014 (Table 1). The first lake, Portles See (POR; 2,892 m a.s.l.), is situated 15 km east of the second one, Rasass See (RAS; 2,682 m a.s.l), and 6 km west of the third one, Oberer Saldursee (SAL1; 2,922 m a.s.l.) (Figure 2). The instrumental temperature records from the nearest meteorological station Marienberg/Montemaria (1,323 m a.s.l.) over the period 1981–2010 (http://www.zamg.acaat/histalp) and the MAAT lapse rate of 0.57°C/100 m for slopes in the Upper South Tyrol region (Rolland, 2003) suggest that the altitudinal position of the −2°C MAAT isotherm is located at ca. 2,800 m a.s.l. The lakes POR and SAL1 and their catchments are thus located within the periglacial belt where frost action conditions dominate (MAAT lower than −2.0°C) (Table 1). RAS and its catchment are located within the periglacial belt where water persists in the liquid phase for most of the year and frost action occurs but does not dominate (MAAT between −2.0°C and −1.4°C). Soil cover is sparse and highly fractured bedrock is exposed in 85%–95% of the catchment areas of the lakes. All three lakes lack surface inflows. POR and RAS are situated at the toes of small active rock glaciers occupying north‐facing slopes, whereas SAL1 is a lake without rock glaciers in its predominantly south‐facing catchment (Figure 2). The proximity to a rock glacier suggests that both, POR and RAS, are closely connected to the internal drainage systems of their adjacent rock glacier. An earlier study of POR and RAS, combining paleoecological techniques, showed that acidic conditions (water pH < 6.5) persisted in both lakes during at least the past two centuries (Psenner & Schmidt, 1992). Our previous investigations yielded evidence demonstrating that ARD in the RAS catchment has been formed within the freshly deglaciated area at the onset of the Holocene (Ilyashuk et al., 2014).

**Table 1:** Selected characteristics of the lakes and their catchments

Parameter	Lake		
	RAS	POR	SAL1
*Lake*			
Latitude (N)	46°44′50″	46°44′50″	46°45′24″
Longitude (E)	10°27′23″	10°38′49″	10°43′17″
Elevation (m a.s.l.)	2,682	2,892	2,922
Mean summer air temperature (°C)^a^	5.9	4.6	4.4
Mean annual air temperature (MAAT, °C)^a^	−1.4	−2.6	−2.7
Surface area (ha)	1.5	0.7	0.6
Maximum depth (m)	9.3	13.5	9.0
*Lake catchment*			
Lake catchment area (ha)	22	12	7.0
Rock glacier/catchment area (%)	18.5	30.0	0
Rock glacier elevation (m a.s.l.)	2,700–2,800	2,892–3,040	—
MAAT range for rock glacier elevations (°C)^a^	−1.4 to −2.0	−2.6 to −3.4	—
*Elemental composition of bedrock* b			
Si (mg/g DW; [282])	242 ± 21	272 ± 39	261 ± 57
Al (mg/g DW; [82.3])	102 ± 20	93 ± 22	97 ± 27
Fe (mg/g DW; [56.3])	50 ± 0.3	41 ± 8	38 ± 10
Ca (mg/g DW; [41.5])	16 ± 2	11 ± 2	10 ± 3
Mn (μg/g DW; [950])	597 ± 2	696 ± 72	594 ± 81
S (μg/g DW; [350])	590 ± 10	545 ± 62	568 ± 46
Zn (μg/g DW; [70])	90 ± 2	85 ± 13	76 ± 30
Ni (μg/g DW; [84])	24 ± 6	50 ± 17	43 ± 11
Cu (μg/g DW; [60])	<20	30 ± 12	25 ± 8

a The mean values are based on instrumental weather observations at the nearest meteorological station Marienberg/Montemaria (1323 m a.s.l.) over the period 1981–2010 (http://www.zamg.acaat/histalp) and corrected for the elevations of the lakes by applying the yearly and summer mean air temperature lapse rates for slopes in the Upper South Tyrol region (Rolland, 2003).

b Values (mean ± *SD*;* n *=* *4) for the chemical composition of the bedrock within the POR and SAL1 catchment are obtained in the present study, and for the RAS catchment are from Sonnleitner et al. (2011); the average abundances of the chemical elements in the Earth's continental crust (Lide, 2004) are given in square brackets.

### 2.2 Water chemistry

Lake water samples were collected during ice‐free seasons at the same time as surface sediment and invertebrate samples. Water samples were taken with a Patalas‐Schindler sampler (UWITEC Ltd., Austria) at 2‐m intervals along a vertical profile in each lake. The physicochemical parameters of water, such as conductivity, pH, alkalinity, and concentrations of major ions and nutrients, were determined following standard methods and analytical quality control procedures (Mosello & Wathne, 1997; The MOLAR Water Chemistry Group, 1999). Total concentrations of trace elements in water samples were determined by inductively coupled plasma‐optical emission spectrometry (ICP‐OES) with standard addition methods (Boss & Fredeen, 2004). The free ion concentrations of aluminum (Al^3+^), manganese (Mn^2+^), and nickel (Ni^2+^) in lake water samples were calculated from their total concentrations measured by applying the visual minteq ver. 3.1 thermodynamically based speciation model (Gustafsson, 2016) with the input of measured lake water characteristics: temperature, pH, DOC, Ca^2+^, Mg^2+^, Na^+^, K^+^, Cl^−^, NO${}_{3}^{-}$, NH${}_{4}^{+}$, SO${}_{4}^{2-}$, Ba, Co, Cu, Fe, Sr, and Zn. The Stockholm Humic Model (Gustafsson, 2001) was used in the model to estimate the effect of DOC on metal speciation.

### 2.3 Catchment bedrock, lake sediments, and invertebrates

For subsequent geochemical analysis, bedrock samples were collected randomly (*n *=* *4) from non‐glaciated parts of the lake catchments. Surface sediment (0–1 cm) samples were obtained from three random sites within the deepest part of the lakes using an Ekman grab sampler with a lining of plastic inside. Typical biotic components of high alpine lakes, the water beetles *Agabus bipustulatus* (Coleoptera: Dytiscidae), and larvae of the chironomid midge *Pseudodiamesa nivosa* (Diptera: Chironomidae), were collected for multi‐element analysis of their body tissues. The chironomid *P. nivosa* shows a 1‐year life cycle (Lindegaard, 1992; Tátosová & Stuchlík, 2006), whereas the beetle *A. bipustulatus* displays a 2‐year life cycle (Nilsson & Holmen, 1995) in cold environments. Adult individuals of *A. bipustulatus* were collected with a kick net in the shallow‐water habitats of the lakes. Larvae of the chironomid *P. nivosa* were collected with a biologic bottom dredge (EFE & GB Nets, UK) in the deep‐water habitats of the lakes. Invertebrates were transported to the laboratory where they were placed in Petri dishes and kept at 4°C for 24 hr to purge the guts. Thereafter, they were triple rinsed with deionized water.

All samples were dried until constant dry weight at 40°C in a laboratory furnace and milled into powder with an agate ball mill. Elemental composition of the rock and sediment samples (fine‐grinded powder in Chemplex SpectroMicro^®^ Sample Cups 3110) and the invertebrate samples (felt‐like aggregates in Chemplex SpectroMicro^®^ Sample Cups 3110 with 3 μm‐thick Prolene films) was assayed by energy dispersive X‐ray fluorescence (EDXRF) analysis using a Spectro XEPOS Plus spectrometer and calibration models based on the fundamental parameter methods (Tessadri, 2003). The experimental parameters were obtained by calibration of the whole system by means of the CCRMP reference soils SO‐1, SO‐2, SO‐3, and SO‐4 (Steger, Bowman, & McKeague, 1985). Results obtained on five replicate samples demonstrate that the relative standard deviation on the metal assay is better than ±10%. The EDXRF analysis is an accurate, precise, and reliable analytical technique widely used in a variety of fields (West et al., 2007).

### 2.4 Sediment coring and chronological control

Sediment cores were obtained with a gravity corer (UWITEC Ltd., Austria) from the deepest part of each lake. The RAS and SAL1 cores dominated by organic‐rich sediments were sectioned at 0.22‐cm increments. The POR sediment core with a higher proportion of clay and sand was sectioned contiguously into 0.5 cm thick samples. Chronological control for the POR sediment sequence was provided by four accelerator mass spectrometry (AMS) radiocarbon dates derived from terrestrial plant macrofossils. The age‐depth model developed for the RAS sediment sequence (Ilyashuk et al., 2014) was revised for the top 16 cm of sediment in the light of two additional AMS radiocarbon dates and the ^210^Pb‐derived ages (see Appendix S1 and Figure S1 for further details). The age‐depth model developed for the SAL1 core has been described in detail by Ilyashuk, Ilyashuk, Tylmann, Koinig, and Psenner (2015). The sediment sequences covering the last millennium were only considered in the present study.

The multi‐proxy summer air temperature reconstruction based on tree‐ring and lake sediment (biogenic silica flux, chironomids) data from the Greater Alpine Region (43–49°N and 4–19°E) (Trachsel et al., 2012) was used for the identification of periods of cold conditions responsible for reduced oxidation of the sulfide minerals and weakened ARDs in the past millennium.

### 2.5 Morphological abnormalities in chironomids

The incidence of morphological abnormalities in chironomid larvae was used for assessing patterns and temporal trends in the ecotoxicological state of the lakes over the past millennium. Larval head capsules of the chironomid *Pseudodiamesa nivosa* found in both dredge and sediment core samples (live specimens and their subfossil remains, respectively) from all three lakes were inspected for deformities of the mentum, a mouth part which is typically well preserved in subfossil material. The *P. nivosa* head capsules were inspected for the presence of mentum gaps, distinct deep smooth‐edged indentations within the mentum, following a recent study by Salmelin, Vuori, and Hämäläinen (2015) that demonstrated that a more sensitive detection of effects of environmental toxicity on chironomids can be obtained by taking into account only the most obvious mentum deformities such as missing and extra teeth and/or mentum gaps. The head capsules were extracted from the sediment core samples following the standard procedure described by Brooks, Langdon, and Heiri (2007). The menta damaged during the cleaning and mounting process usually are easily distinguishable from deformed structures (Dermott, 1991). A minimum of 100 fourth‐instar head capsules of *P. nivosa* were inspected for mentum gaps in each sample at up 250× magnification using a compound microscope. Stratigraphic diagrams were produced with the software packages Tilia (Grimm, 2011).

### 2.6 Statistical analyses

One‐way analysis of variance (ANOVA), followed by Tukey's post hoc test, was used to determine whether there are any significant differences in the elemental composition of water, sediment, and biota between the lakes. The nonparametric Mann–Whitney *U‐*test was used to detect significant differences in the frequency of mentum gaps between *P. nivosa* populations of the lakes. All data were square root transformed to reduce the biasing effect and stabilize variances. All statistical analyses were performed using the spss software package (SPSS Inc., Chicago, IL, USA).

## 3 RESULTS

### 3.1 Bedrock geochemistry

Chemical analysis of rock samples showed that the geochemical composition of bedrock is rather similar between the lake catchments (Table 1). The catchments are characterized by elevated sulfur concentrations compared to its Earth crustal abundance (Lide, 2004). The concentrations of most metals are lower or comparable with their abundances in the Earth's continental crust.

### 3.2 Lake water chemistry

Chemical analysis of water samples revealed that although the water in all three lakes is dominated by the same ions (Ca^2+^, Mg^2+^, and SO${}_{4}^{2-}$), the total ion concentrations differ strongly between the lakes. The electrical conductivity of water in RAS is 1.6 times higher than in POR and five times higher than in SAL1 (Table 2). The total concentrations of aluminum, manganese, nickel, and zinc are negligibly small in the circumneutral SAL1 (pH ~7.7; without rock glaciers in the catchment) compared to the acidic RAS (pH ~5.2) and POR (pH ~5.4) situated at the toes of rock glaciers. The total concentrations of manganese and nickel exceed the appropriate EU limits for drinking water (The Council of the EU, 1998) ~16 and ~8 times, respectively, in the POR water and **˜**23 and ~15 times, respectively, in RAS (Table 2). The total concentrations of aluminum in RAS and POR exceed the appropriate limit for drinking water more than four times. The sulfate concentrations in RAS are equal to the EU maximum permissible levels in drinking water, but lower in POR and much lower in SAL1.

**Table 2:** Physical and chemical properties of water in the lakes in the ice‐free season

	Lake			
Parameter	RAS (*n *=* *8)	POR (*n *=* *5)	SAL1 (*n *=* *7)	EU limit value
Conductivity (μS/cm at 25°C)	566 ± 125	346 ± 1	112 ± 1	2,500
Total ions (μequiv./L)	11,775 ± 2,194	6,337 ± 49	1,977 ± 17	NA
Ca^2+^ (mg/L)	49 ± 11^a^	34 ± 1^a^	14 ± 1	NA
Mg^2+^ (mg/L)	26 ± 7^a^	17 ± 1^a^	3 ± 1	NA
Na^+^ (mg/L)	2.3 ± 0.6^a^	1.4 ± 0.1^a^	0.5 ± 0.1	200
K^+^ (mg/L)	0.8 ± 0.1^a^	1.1 ± 0.1^a^	0.5 ± 0.1^a^	NA
SO${}_{4}^{2-}$ (mg/L)	**248 ± 34**	150 ± 1	35 ± 1	250
P_tot_ (μg/L)	1.5 ± 0.1^a^	1.2 ± 0.1^a^	1.9 ± 0.3^a^	NA
DOC (μg/L)	252 ± 25	159 ± 5	342 ± 7	NA
Al_tot_ (μg/L)	**1,000 ± 211** ^a^	**866 ± 16** ^a^	<1	200
Cu_tot_ (μg/L)	6 ± 2^a^	4 ± 1^a^	<1	2,000
Fe_tot_ (μg/L)	4 ± 2	<1	<1	200
Mn_tot_ (μg/L)	**1,167 ± 154**	**826 ± 14**	<1	50
Ni_tot_ (μg/L)	**299 ± 58**	**158 ± 1**	<1	20
Sr_tot_ (μg/L)	181 ± 41	56 ± 1	29 ± 3	NA
Zn_tot_ (μg/L)	207 ± 41	110 ± 1	<1	NA
pH (units)	**5.22 ± 0.07**	**5.43 ± 0.01**	7.71 ± 0.05	≥6.5

RAS data for 2011–2012, POR for 2014, and SAL1 for 2010–2012. For each parameter, values (mean ± *SE*) sharing a letter in their superscript do not differ significantly (one‐way ANOVA and Tukey's post hoc test, *p *<* *.05). DOC = dissolved organic carbon; NA = not available, that is parameter is not regulated by the European Drinking Water Directive (The Council of the EU, 1998); parameter values equal to or exceeding the appropriate European Union limit values for drinking water are shown in bold.

Speciation analysis performed with the visual minteq equilibrium model demonstrated that free Ni^2+^ and Mn^2+^ ions account for 88%–91% of the total nickel and manganese concentrations in RAS and POR (Table 3). Free Al^3+^ ions account for 20%–23% of the total aluminum concentrations in both lakes.

**Table 3:** Free ion concentrations (mean ± *SD*) of aluminum (Al^3+^), manganese (Mn^2+^), and nickel (Ni^2+^) and their proportions among dissolved species calculated with the Visual MINTEQ chemical equilibrium model in water of the lakes RAS (*n *=* *8) and POR (*n *=* *5)

Component	Concentration (μmol/L)	Mean percentage of total concentration
*RAS*		
Al^3+^	5.9 ± 0.7	20
Mn^2+^	17.1 ± 2.5	90
Ni^2+^	4.1 ± 0.6	89
*POR*		
Al^3+^	7.5 ± 0.3	23
Mn^2+^	13.6 ± 0.5	91
Ni^2+^	2.4 ± 0.1	88

### 3.3 Geochemistry of surface sediments

**Figure 3:**
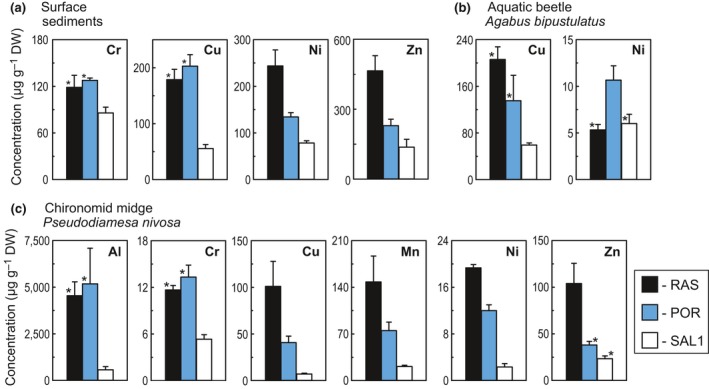
Total concentrations (mean ± *SD*;* n *=* *3) of selected elements in (a) surface sediments from the deepest areas and (b and c) biota of the high alpine lakes Rasass See (RAS), Portles See (POR), and Oberer Saldursee (SAL1). For each chemical element, aluminum (Al), chromium (Cr), copper (Cu), manganese (Mn), nickel (Ni), and zinc (Zn) values marked with asterisks do not differ significantly (one‐way ANOVA, Tukey's post hoc test, *p *<* *.05) between the lakes [Colour figure can be viewed at wileyonlinelibrary.com]

The total concentrations of chromium, copper, nickel, and zinc in the deep‐water sediments are significantly greater (one‐way ANOVA, Tukey's post hoc test, *p *<* *.05) in RAS and POR, the lakes with the rock glaciers in the catchments, than in SAL1 (Figure 3a). It is noteworthy that nickel and zinc concentrations are significantly less (one‐way ANOVA, Tukey's post hoc test, *p *<* *.05) in POR whose catchment is governed mainly by frost action, compared to RAS, lying in the catchment dominated by melting of ground ice in the rock glacier.

### 3.4 Invertebrate body burdens

The metal body burdens vary substantially among the studied lakes and invertebrate taxa (Figure 3b,c). Concentrations of five metals (Al, Cr, Cu, Mn, and Ni) in the chironomid *Pseudodiamesa nivosa* are significantly greater (one‐way ANOVA, Tukey's post hoc test, *p* < .05) in RAS and POR as compared to SAL1. The greatest concentrations of manganese, nickel, and zinc were measured in *P. nivosa* from RAS. Similarly, the highest copper body burdens were found in the water beetle *Agabus bipustulatus* from RAS.

### 3.5 Morphological abnormalities in chironomids: the present and past

**Figure 4:**
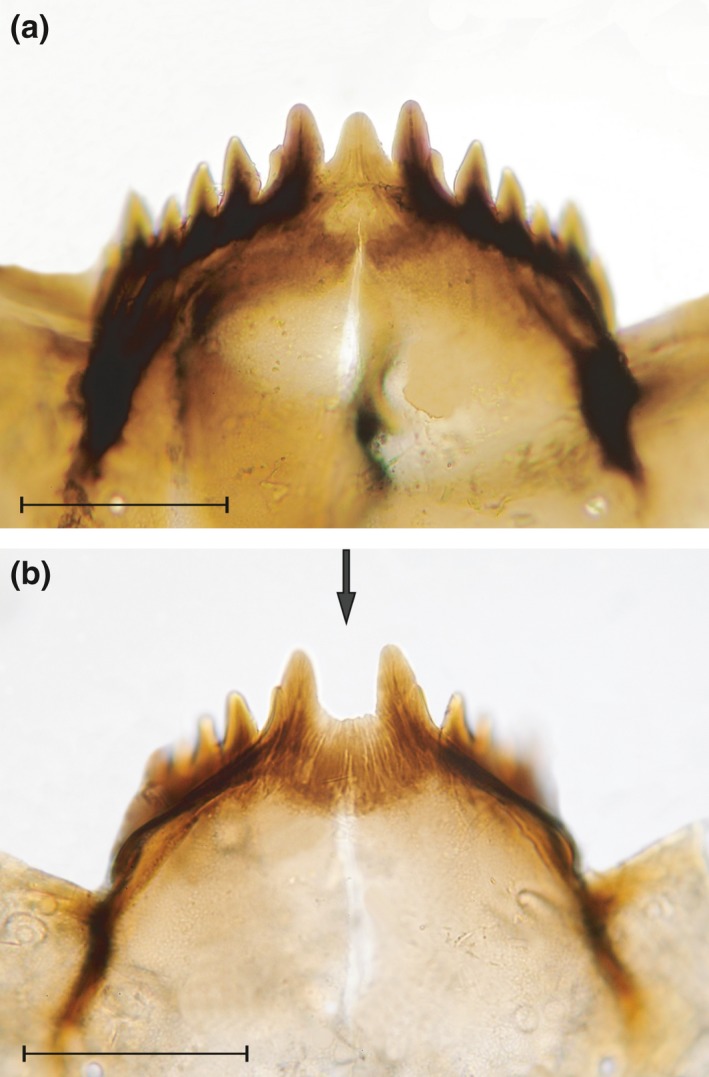
Photomicrographs (200× magnification) of larval menta of the chironomid *Pseudodiamesa nivosa* from the Portles See sediment core: (a) normal mentum and (b) deformed mentum with deep smooth‐edged gap. Scale bars = 0.1 mm [Colour figure can be viewed at wileyonlinelibrary.com]

The inspection of contemporary *Pseudodiamesa nivosa* larvae, represented by live and subfossil specimens in surface sediments, for mentum abnormalities revealed a high incidence of mentum gaps (Figure 4) in the RAS and POR populations. In RAS, the frequency of mentum gaps varies from 30% to 38% (*n *=* *3) in the live larvae and is comparable to that of their recent remains (32%–38%, *n *=* *3). The incidence of mentum gaps varies from 9% to 15% (*n *=* *3) in the live larvae and from 7% to 14% (*n *=* *3) among their subfossils in POR. In SAL1, without a rock glacier in the catchment, the frequency of mentum gaps is not higher than 1% (*n *=* *3) among both contemporary and subfossil specimens of *P. nivosa*, which is well within natural background levels for chironomid populations (Vermeulen, 1995).

**Figure 5:**
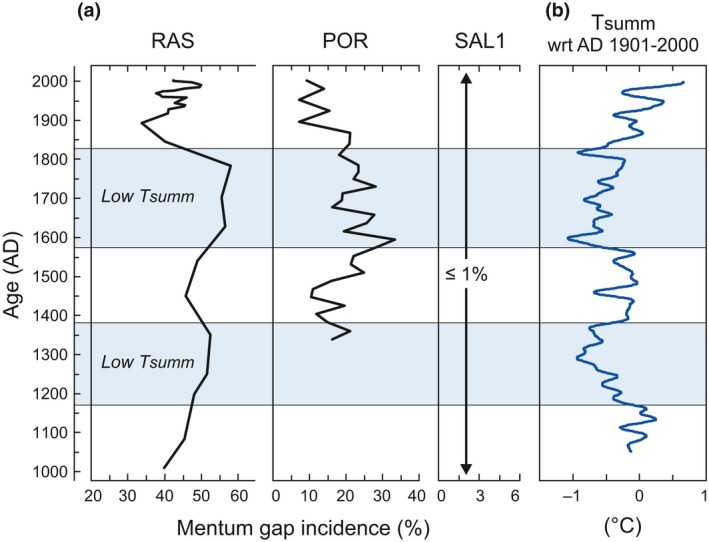
Mentum gap incidence in populations of the midge *Pseudodiamesa nivosa* in the sediment cores from Rasass See (RAS), Portles See (POR), and Oberer Saldursee (SAL1) plotted against summer air temperature (Tsumm) anomalies (compared to the mean of the 20th century) reconstructed from paleoclimate multi‐proxy archives in the European Alps (Trachsel et al., 2012); the shaded areas correspond to the periods (AD 1172–1379 and AD 1573–1822) of low Tsumm during the Little Ice Age [Colour figure can be viewed at wileyonlinelibrary.com]

The analysis of subfossil *P. nivosa* head capsules in down‐core sediment samples showed that a high mentum gap incidence, between 33% and 58%, persisted in the RAS population throughout the past millennium (Figure 5). The frequency of mentum gaps was significantly lower (7%–33%; Mann–Whitney *U‐*test, *p *<* *.0003) in the POR population of *P. nivosa* over the investigated time interval spanning the past seven centuries, and did not exceed 1% in the SAL1 population during the past millennium. The highest rates of mentum gap incidence in the stressed *P. nivosa* populations of RAS (55%–58%) and POR (16%–33%) were recorded during the LIA, the coldest period over the last millennium in the European Alps (ca. AD 1570–1820; Trachsel et al., 2012) (Figure 5). The rates of mentum gap incidence in RAS and POR during the LIA are approximately 1.4 and 2.3 times higher (Mann–Whitney *U‐*test, *p *<* *0.002), respectively, than over the post‐LIA period.

## 4 DISCUSSION

The lake water and sediment geochemical data (Table 2 and Figure 3a) provide evidence for the presence of natural ARDs in the catchments of the lakes situated at the toes of rock glaciers and suggest that POR and its catchment located in the periglacial environment with a MAAT below −2°C are affected by less severe ARD than RAS and its catchment located within the periglacial belt with MAATs ranging from −2°C to +3°C. Under more acidic conditions in RAS, the total concentrations of many minor and trace elements (Mn, Ni, Sr, and Zn) in the lake water are significantly greater than in POR. This may be explained by the fact that the rates of chemical and microbiological processes interacting in oxidative leaching of the sulfide‐bearing bedrock are temperature‐dependent and increase with temperature (Ahonen & Tuovinen, 1991; Chandra & Gerson, 2010; Fan et al., 2016). Temperature alone, however, can play less of a role in regulating chemical weathering of rock minerals than the availability of water (Egli, Wernli, Kneisel, & Haeberli, 2006; Egli et al., 2015; Maher & Chamberlain, 2014). Consequently, the oxidative weathering of sulfide minerals, which is responsible for ARD generation, may proceed more intensively in periglacial environments where water remains in the liquid state for a longer period of time and extensive fresh rock surfaces are subjected to more prolonged exposure to air and oxygenated water. As a rule, high concentrations of dissolved weathering products, which are typical for rock glacier meltwater (Fegel, Baron, Fountain, Johnson, & Hall, 2016; Lecomte, Milana, Formica, & Depetris, 2008; Williams, Knauf, Caine, Liu, & Verplanck, 2006), decrease the freezing point of water by a few degrees (Elberling, 2001; Williams & Smith, 1989) and favor the exposure of fresh rock surfaces to oxygen. Under ambient temperatures above or close to the freezing point, permafrost degradation leads to high macro‐porosity (cracks, channels) of rock glacier deposits and increased water content, more than 45%, in their base layers (Hauck, Böttcher, & Maurer, 2011; Ikeda, Matsuoka, & Kääb, 2008; Monnier & Kinnard, 2013). Our results suggest that ambient temperature is an important factor that affects ARD production within rock glaciers both directly and indirectly, through its effect on the deposit porosity and unfrozen water content, leading to water quality deterioration in periglacial areas.

The influx of ARD into a lake can severely degrade both habitat and water quality producing an unfavorable environment for aquatic life (Luoma & Rainbow, 2008). High metal body burdens observed in the invertebrates from RAS and POR reflect extremely adverse environmental conditions in these lakes affected by the ARDs, in contrast to the reference lake SAL1. The difference between the lakes in the metal body burdens observed in the invertebrates reflects the difference in the presence as well as in the severity of ARD. Bervoets, De Jonge, and Blust (2016), who related metal body burdens in the larval chironomid *Chironomus* sp. to water quality indices based on benthic invertebrate community metrics, estimated safe threshold body burdens for chromium, copper, and nickel of 10, 57, and 6.5 μg/g DW, respectively, above which good ecological quality of surface waters is never reached. Findings from our study reveal that in larvae of the chironomid *Pseudodiamesa nivosa* from RAS, these thresholds for Cr, Cu, and Ni are exceeded by 1.2, 1.8, and 2.9 times, respectively, and in the *P. nivosa* larvae from POR, the thresholds for Cr and Ni are exceeded by 1.2 and 1.8 times, respectively, providing one more indication of environmental adversity.

The total concentrations of aluminum, manganese, and nickel in RAS and POR exceed the appropriate EU limits for drinking water up to more than one order of magnitude. Among these elements, aluminum generally is not as highly toxic to aquatic invertebrates as manganese and nickel (Gensemer & Playle, 1999). Manganese was often regarded as one of the least toxic elements, but more recent studies have shown that exposure to high concentrations of manganese can induce cytotoxic and genotoxic effects and morphological alterations in invertebrates (Nicosia, Salamone, Mazzola, & Cuttitta, 2015; Pinsino, Matranga, & Roccheri, 2012). Nickel is also known as a cytotoxic (Blewett & Leonard, 2017; Leonard & Wood, 2013) and genotoxic (Dallas, Bean, Turner, Lyons, & Jha, 2013) agent for cells of aquatic invertebrates. Bioavailability and toxicity of waterborne metals to aquatic organisms generally depend on the concentration and activity of free metal ions, which is controlled by various chemical speciation processes (Hare & Tessier, 1996; Morel & Hering, 1993). Despite some differences in water chemistry of RAS and POR, these processes lead to similar proportions of the total dissolved metals present as free Al^3+^, Mn^2+^, and Ni^2+^ ions in both lakes and the differences in the concentration of these free ions between the lakes are equivalent to those that are recorded in their total concentrations.

An elevated incidence of morphological deformities in chironomid larvae is generally caused by disrupting physiological processes during larval ontogeny, as a somatic response to xenobiotic substances (Groenendijk, Zenstra, & Postma, 1998; Vermeulen, Liberloo, Ollevier, & Goddeeris, 2000). The results of paleoecological investigation of the frequency of mentum gaps in the *Pseudodiamesa nivosa* populations of the lakes suggest that harmful conditions in POR and much more adverse conditions for aquatic life in RAS persisted throughout the past millennium. These findings provide evidence that the ARDs in the catchments of these lakes are of a long‐lasting nature and cannot be associated with only the temperature increase over recent decades.

The frequency of mentum deformities in the stressed *P. nivosa* populations of RAS and POR was significantly higher during the LIA than during pre‐ or post‐LIA periods, rejecting our hypothesis that more favorable conditions for aquatic life can be expected in the lakes during the LIA due to reduced sulfide mineral oxidation and reduced release of ARD contaminants generally observed at lower air temperatures (cf. Ahonen & Tuovinen, 1991; Chandra & Gerson, 2010; Fan et al., 2016; Schoonen et al., 2000). This may initially seem somewhat surprising. However, several studies based on data from present‐day freshwater ecosystems polluted by urban and industrial wastes have shown that the frequency and severity of morphological deformities in chironomid larvae vary seasonally, with higher values observed during cold periods and lower values during warm periods of the year (Servia, Cobo, & González, 2000; van Urk, Kerkurm, & Smit, 1992; Vermeulen, 1998). Maximum frequencies were observed in the overwintering generations of chironomid larvae (van Urk et al., 1992). These results are fully consistent with our findings of increased deformity frequency in colder periods of the past millennium. This phenomenon, according to the “time‐of‐exposure” hypothesis proposed by Vermeulen (1998) and Goddeeris et al. (2001), can reflect indirect effects of temperature, mediated mainly through the influence on the developmental rate of individuals, on the morphological alteration in chironomid larvae. Slower growth and development of chironomid larvae at low temperatures result in more prolonged exposure to toxic substances during the cold season in comparison with larvae that develop during the warm season (Servia, Cobo, & González, 2004; Servia et al., 2000). It is likely that prolonged ice cover on lakes during the LIA cooling (Brown & Duguay, 2010) was one of the key factors determining the duration of larval exposure to toxicants.

It is generally recognized that the cooling events accompanied by prolonged ice cover on lakes may have resulted in extensive hypolimnetic oxygen depletion (Leppäranta, 2015). Under anoxic conditions, however, many metals (e.g. Cd, Cu, Ni, and Zn) may be less soluble than in the presence of oxygen (di Toro et al., 1992; Wright & Welbourn, 2002). Elevated oxygen concentrations in overlying surface water mediated through shortened ice seasons, on the contrary, can directly enhance the leaching of sediment‐bound metals to water and metal toxicity to aquatic invertebrates (De Jonge, Teuchies, Meire, Blust, & Bervoets, 2012a, b). As a rule, prolonged ice cover on lakes is also accompanied by a decrease in lake water pH, one more factor affecting metal partitioning and speciation and their toxicity to aquatic organisms, caused by CO_2_ oversaturation of water during winter (Wögrath & Psenner, 1995). Decreased levels of lake water pH in RAS and POR were reconstructed for the cold LIA period in the earlier study by Psenner and Schmidt (1992). Lowering water pH prevents the transfer of most trace metals from the water phase to the sediment and causes a desorption from the sediments, that results in increased concentrations of free metal ions (Salomons, 1995). However, studies with chironomids and other aquatic organisms have shown a decrease in metal toxicity with decreasing pH as a result of increased competition between metal ions and H^+^ (Peck, Klessa, & Baird, 2002; Wilde, Stauber, Markich, Franklin, & Brown, 2006). Consequently, hypolimnetic oxygen depletion and lowered water pH associated with prolonged ice cover cannot explain higher metal toxicity and frequency of chironomid deformities in the studied lakes during the LIA.

Exposure duration, temperature, and metal toxicity to aquatic invertebrates have been the focus of many recent ecotoxicology studies. On the one hand, a number of studies evaluating the toxicity of waterborne metals to invertebrates under acute exposure (over ca. 24–72 hr to high metal concentrations) have indicated that acute metal toxicity increases with the increase in temperature (Boeckman & Bidwell, 2006; Ferreira, Serra, Soares, & Loureiro, 2010; Mo, Yoo, Bae, & Cho, 2013; Soares, Cativa, Moreira‐Santos, Soares, & Ribeiro, 2005; Vedamanikam & Shazili, 2009). On the other hand, a more recent study investigating the effect of temperature on metal toxicity under chronic exposure (over at least 10% of an organism's lifespan to lower metal concentrations), which may be a more realistic scenario relevant to our lakes (cf. Suter, 1993), demonstrates that chronic metal toxicity to invertebrates can increase with the decrease in temperature (Pereira, Deruytter, Blust, & De Schamphelaere, 2017). Consequently, the effect of temperature on metal toxicity to aquatic invertebrates can differ between these two exposure scenarios. The processes of detoxification and elimination of metals, in turn, are also temperature‐dependent and could be reduced by low temperatures (Cedergreen et al., 2013; Pereira et al., 2017). In general, these studies emphasize that the relationship between metal toxicity and temperature is not always straightforward because of other factors involved. In natural ecosystems, where a combination of physical, chemical, and biologic factors may significantly alter metal bioavailability and toxicity to aquatic invertebrates, these relationships are far more complex. Our paleoecological inferences from the lakes located in the periglacial belt are in line with the findings of Pereira et al. (2017) in the chronic toxicity experiments, which showed that lower temperatures can increase metal toxicity to invertebrates exposed to toxicants for a long time. Apparently, the temperature‐mediated effects of exposure duration and physiological mechanisms of the chironomid response amplify the mentum gap incidences in the RAS and POR populations during cold periods, accompanied by prolonged lake ice cover and slowed down growth and development of the chironomids.

Thus, the higher frequency of chironomid morphological deformities in both ARD‐stressed lakes during the LIA (Figure 5) indicates that lower water temperatures and prolonged ice cover may have been the key factors increasing the adverse impacts of ARD on aquatic invertebrates despite an expected reduced release of ARD contaminants under cooler air conditions. Given that the oxidation rate of sulfide minerals increases with temperature (e.g., Ahonen & Tuovinen, 1991; Schoonen et al., 2000), a deterioration of conditions for aquatic life can be expected in the lakes at higher temperatures. However, the decrease in the incidence of abnormalities at the temperature increase of ~1°C during the termination of the LIA (AD ~1820–1900; Figure 5) suggests that effects related to a lower chronic metal toxicity to invertebrates at higher temperatures were more significant in both lakes than effects related to enhanced oxidation of sulfide minerals and ARD production in the catchments. In the post‐LIA period, the incidence of chironomid abnormalities has persisted at a low level since AD ~1900 in POR, the lake with a catchment located at an elevation that constantly remained above the −2°C isotherm of MAAT. In contrast, the abnormality incidence began to rise in RAS, the lake with a catchment located at an elevation that certainly crossed the threshold MAAT value of −2.0°C at the termination of the LIA, thus allowing for increasing ARD production. This increase in ARD seems to have had a stronger impact on chironomids than compensatory effects related to a decrease in chronic metal toxicity to invertebrates at higher temperatures. Our investigation of the incidence of morphological abnormalities in chironomids over time shows that climate‐mediated effects on the metabolism and life cycle of aquatic organisms should be taken into account when reconstructing long‐term temporal trends in the ecotoxicological state of lakes. Altogether, the results of the paleolimnological analysis allowed us to track the periods of more unfavorable conditions for aquatic life in the ARD‐stressed lakes, highlighting that examining chironomid mentum deformities is a valid paleo‐ecotoxicological tool.

Overall, the present study focuses on extreme ARD areas associated with rock glaciers and their effects on headwater lakes in periglacial environments, rather than on rock glacier thawing per se. Findings based on contemporary ecological and paleolimnological approaches in our study are of equal importance and complement one another. There may be some concern about the limited numbers of lakes included in the study. However, the lakes selected for the comparative investigation provided a unique model to test our hypotheses. Both ARD‐stressed lakes are situated at the toes of the active rock glaciers, that is, they have very close connections with internal drainage systems of the rock glaciers. Their catchments located below and above the threshold MAAT value of −2.0°C for the periglacial environments are just within a radius of *˜*10 km, which minimizes the differences in underlying bedrock geology and annual precipitation amounts. Changes along these and other environmental gradients may also affect the severity of ARD (Jacobs, Lehr, & Testa, 2014). While the immediate vicinity of a lake to a rock glacier is quite rare, it would be useful to test our hypotheses and obtained results on a broader set of lakes and in different mountain areas of the world. Additionally, investigations of internal structure and dynamics of rock glaciers may help to extend and elaborate our knowledge and understanding of the ARD phenomenon in pristine periglacial environments. Thus, many important questions await future investigation to improve our knowledge about permafrost‐related threats to alpine headwater lakes.
