# Supplementary material for: Rock glaciers in crystalline catchments: Hidden permafrost‐related threats to alpine headwater lakes
Source: Glob Chang Biol. 2017 Dec 4;24(4):1548–62. doi: 10.1111/gcb.13985 (PMC5873409; doi:10.1111/gcb.13985)
Supplement: Supplementary file 1 [file GCB-24-1548-s001.docx]

**Rock glaciers in crystalline catchments: hidden permafrost-related threats to alpine headwater lakes**

Boris P. Ilyashuk^1, 2^, Elena A. Ilyashuk^1^, Roland Psenner^1, 2^, Richard Tessadri^3^ and Karin A. Koinig^1, 2^

^1^Institute of Ecology, University of Innsbruck, Technikerstraße 25, 6020 Innsbruck, Austria

^2^Institute for Alpine Environment, European Academy Bozen/Bolzano, Viale Druso 1, 39100 Bozen/Bolzano, Italy

^3^Institute of Mineralogy and Petrography, University of Innsbruck, Innrain 52, 6020 Innsbruck, Austria

Correspondence: Boris P. Ilyashuk, tel. +43 512 50751736, fax: + 43 512 50751799; e-mail: boris.ilyashuk@uibk.ac.at

**Supporting Information**

**Appendix S1: Sediment Dating and Age-Depth Modeling**

Chronological control for the POR sediment sequence was provided by four accelerator mass spectrometry (AMS) radiocarbon dates derived from terrestrial plant macrofossils. The age-depth model developed for the RAS sediment sequence (Ilyashuk *et al.*, 2014) was revised for the top 16 cm of sediment in the light of two additional AMS radiocarbon dates and the ^210^Pb-derived ages. Radiometric dating of sediments via the measurement of ^210^Pb, ^137^Cs and ^226^Ra radioisotopes was performed using gamma-ray spectrum analysis (Appleby, 2001) at the University of Liverpool. The ^210^Pb-based ages of recent sediments were calculated by applying the constant rate of supply (CRS) model to the unsupported ^210^Pb inventory (Appleby & Oldfield, 1978). The AMS radiocarbon dating of terrestrial plant macrofossils was carried out at the Poznan Radiocarbon Laboratory, Poland. Calibration of ^14^C dates was undertaken with the Bayesian calibration program OxCal 4.2 (Bronk Ramsey, 1995, 2009) by applying the Northern Hemisphere terrestrial IntCal13 calibration curve (Reimer *et al.*, 2013). The radioisotope dates were introduced into the OxCal 4.2 software and the age-depth relationships were established by means of Poisson mediated deposition models (P Sequence, k=2) (Bronk Ramsey, 2008; Bronk Ramsey & Lee, 2013). The final age-depth models were constructed by applying linearly interpolating between the midpoints of the highest posterior density range of modeled dates (Figure S1).

The chronology of the SAL1 sediment core was based on five AMS radiocarbon dates and the ^210^Pb-based ages for the top 12 cm of the core. The age-depth model developed for the SAL1 core has been described in detail by Ilyashuk *et al.* (2015).


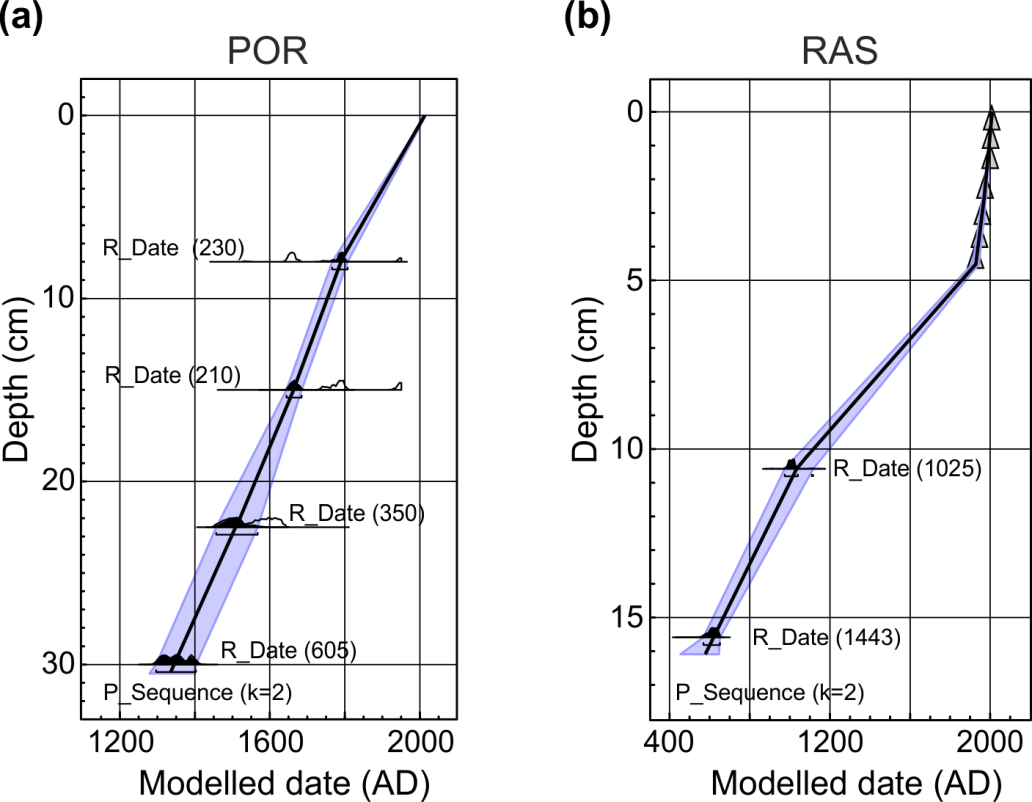


**Fig. S1** Age-depth relationships for the sediment cores from (a) Portles See (POR) and (b) Rasass See (RAS). The relationships are based on Bayesian statistics for ^14^C (R_Dates) and ^210^Pb (grey triangles, RAS) dates. The plots show 2-sigma ranges of the prior likelihood distributions of each calibrated radiocarbon date, the 95% confidence intervals (shaded areas) for the fitted models and the complete age-depth curves (thick lines) constructed by linearly interpolating between the midpoints of the highest posterior density range of all calendar ages.

**Supplementary References**

Appleby PG (2001) Chronostratigraphic techniques in recent sediments. In: *Tracking Environmental Changes Using Lake Sediments, Vol. 1: Basin Analysis, Coring and Chronological Techniques* (eds Last WM, Smol JP), pp. 171–204. Kluwer Academic Press, Dordrecht, The Netherlands.

Appleby PG, Oldfield F (1978) The calculation of ^210^Pb dates assuming a constant rate of supply of unsupported ^210^Pb to the sediment. *Catena*, **5**, 1–8.

Bronk Ramsey C (1995). Radiocarbon calibration and analysis of stratigraphy: The OxCal program. *Radiocarbon*, **37**, 425–430.

Bronk Ramsey C (2008) Deposition models for chronological records. *Quaternary Science Reviews*, **27**, 42–60.

Bronk Ramsey C (2009). Bayesian analysis of radiocarbon dates. *Radiocarbon,* **51**, 337–360.

Bronk Ramsey C, Lee S (2013) Recent and planned developments of the program OxCal. *Radiocarbon*, 55, 720–730.

Ilyashuk BP, Ilyashuk EA, Psenner R, Tessadri R, Koinig KA (2014) Rock glacier outflows may adversely affect lakes: lessons from the past and present of two neighboring water bodies in a crystalline-rock watershed. *Environmental Science and Technology*, **48**, 6192**–**6200.

Ilyashuk EA, Ilyashuk BP, Tylmann W, Koinig KA, Psenner R (2015) Biodiversity dynamics of chironomid midges in high-altitude lakes of the Alps over the past two millennia. *Insect Conservation and Diversity,* **8**, 547–561.

Reimer PJ, Bard E, Bayliss A *et al.* (2013) IntCal13 and Marine13 radiocarbon age calibration curves 0–50,000 years cal BP. *Radiocarbon*, **55**, 1869–1887.
